# Predicting Breast Cancer Risk for Women Veterans of African Ancestry in the Million Veteran Program

**DOI:** 10.1089/heq.2023.0011

**Published:** 2023-05-26

**Authors:** Shiuh-Wen Luoh, Jessica Minnier, Hongyu Zhao, Lina Gao

**Affiliations:** ^1^ VA Portland Health Care System, Portland, Oregon, USA.; ^2^ Knight Cancer Institute, Oregon Health & Science University, Portland, Oregon, USA.; ^3^ OHSU-PSU School of Public Health, Portland, Oregon, USA.; ^4^ Department of Biostatistics, Yale School of Public Health, VA Connecticut Health Care System, New Haven, Connecticut, USA.

**Keywords:** breast cancer, biomarkers, risk prediction and African ancestry

## Abstract

Breast cancer is a leading cause of cancer and, therefore, a major health threat for women in the United States and worldwide. We have seen over the years major advances in breast cancer prevention and care. Breast cancer screening with mammography leads to reduction in breast cancer mortality, and breast cancer prevention treatment with antiestrogens results in reduction in breast cancer incidence. More progress, however, is urgently needed for this common cancer that affects 1 in 11 American women in their lifetime. Not all women have the same breast cancer risk. A personalized approach is highly desirable as women with higher breast cancer risk may benefit from more intense breast cancer screening and/or prevention intervention while lower risk women may be spared with the cost, inconvenience, and emotional burden of these procedures. In addition to age, demographics, family history, lifestyle, and personal health, genetics is an important determinant of an individual's risk for breast cancer. Over the past 10 years, advances in cancer genomics identified multiple common genetic variants from population studies that collectively can contribute significantly to an individual's breast cancer risk. The effects of these genetic variants can be summarized as a “polygenic risk score” (PRS). We are among the first groups to prospectively evaluate the performance of these risk prediction instruments among women veterans of the Million Veteran Program (MVP). A 313-variant PRS (PRS313) predicted incident breast cancer for a prospective cohort of European (EUR) ancestry women veterans with an area under the receiver operating characteristic curve (AUC) of 0.622. The PRS313 performed less well for AFR ancestry however, with an AUC of 0.579. This is not surprising as most genome-wide association studies were conducted in people of European ancestry. This is an important area of health disparity and unmet need. The large population size and diversity of the MVP provide a unique and important opportunity to explore novel approaches to produce accurate and clinically useful genetic risk prediction instruments for minority populations.

## Introduction

Breast cancer is a leading cause of cancer death for American women. It is also a serious issue for women veterans. Our most recent Million Veteran Program (MVP) analysis found that out of 53.5K women veterans from the MVP without prior breast cancer diagnosis (mean age at entry 48.8 and 30.0% of African [AFR] ancestry), there were 818 new breast cancer diagnoses over a median follow-up of 5.7 years, translating into a breast cancer incidence of 2.70 per 1000 per year (unpublished).

Breast cancer screening with mammography leads to reduction in mortality from breast cancer.^1^ However, the current breast cancer screening strategy is largely “one size fits all,” except for very high-risk women, such as those with *BRCA* mutations or prior chest wall irradiation. Appropriate risk assessment is highly desirable as higher risk women may benefit from more intense screening approaches, whereas lower risk women may do equally well with less intensity, therefore, avoiding the cost and inconvenience of screening.

In addition, tamoxifen has been approved for women above the age of 35 years for breast cancer prevention if they have an elevated risk, as seen with an average 65-year-old Caucasian woman.^2,3^ There are a growing number of characteristics that have been validated to predict a woman's risk of developing breast cancer. This includes age; mutation status for breast cancer genes, such as *BRCA1* and *BRCA2*; family history of cancer; personal history of benign breast biopsy; history of benign breast disease, such as atypia; exposure to hormones; breast density as seen on mammography; and more recently, background parenchymal enhancement on MRI.^4^

Harnessing these features may enable us to more precisely assess the risk of breast cancer development. Understanding individuals' risks for developing breast cancer may allow us to adopt a more appropriate breast cancer screening and/or prevention strategy. Recent successes from genome-wide association studies (GWAS) of breast cancer (e.g., A study with more than 150,000 breast cancer cases and more than 110,000 controls^5^) have led to a growing literature on the development and evaluation of breast cancer risk prediction models, for example Refs.^6–8^

## Results

In a prospective cohort of 35,130 women veterans without history of breast cancer (median age 49 years and median follow-up 3.9 years) in the MVP,^9^ we evaluated the performance of clinical breast cancer risk prediction models that comprised demographics, lifestyle, personal health, environmental exposure, and a genetic risk model, 313-variant polygenic risk score (PRS313).^10^ The performance of PRS313 alone was assessed. Clinical risk models tested included a literature review and a Breast Cancer Risk Assessment Tool,^11,12^ implemented with or without PRS313. Thirty one percent of this cohort were of non-Hispanic AFR ancestry.

Individualized Coherent Absolute Risk Estimator^11^ literature review^12,13^ in combination with PRS313 had an area under the receiver operating characteristic curve (AUC) of 0.708 (95% confidence interval, 0.659–0.758) in women with European or non-AFR ancestries and 0.625 (0.539–0.711) in AFR women.^9^ Breast Cancer Risk Assessment Tool with PRS313 had an AUC of 0.695 (0.662–0.729) in European or non-AFR women and 0.675 (0.626–0.723) in AFR women.^9^

PRS313 alone without clinical or demographic predictors yielded an AUC of 0.622 (0.580–0.664) in women of European descent and a lower AUC of 0.579 (0.522–0.636) in AFR women. Incorporation of PRS313 with clinical models improved prediction in European, but not in AFR women.^9^ Models estimated up to 9% of European and 18% of AFR women with absolute lifetime risk >20%. Women with a lifetime breast cancer risk of >20% are generally considered to be high risk and may be appropriate for clinical studies that would examine whether these women would benefit from more intense breast cancer screening.

## Discussion

We found that PRS313 underperformed in a prospective cohort of women veterans of AFR ancestry in the MVP. This is consistent with prior cross section or case–control studies.^9,14–16^ This is a significant area of unmet need as African Americans have higher risk of developing early-onset breast cancer and about 40% higher breast cancer mortality than other ancestral groups in the United States.^17^

PRS has shown great promise in improving biomedical outcomes through precision medicine. However, currently available PRS tools are much more accurate in individuals of European descent, as other ancestral groups were under-represented and understudied. In a transethnic breast cancer study,^18^ the authors performed AFR ancestry GWAS meta-analysis (9241 cases and 10,193 controls), and then meta-analyzed with European ancestry GWAS data (122,977 cases and 105,974 controls) from the Breast Cancer Association Consortium. There was a 13-fold difference between the number of breast cancer subjects between European and AFR ancestries.

As a result of sample size and possibly different genetic architecture between the two populations, only six loci were identified from individuals of African ancestry, even after trans-ethnic analysis, in comparison with more than 200 loci reported from individuals of European ancestry. The differential performance of PRS across ancestral groups raises a significant concern about potentials to exacerbate health disparities and evokes ethical controversy surrounding the clinical implementation of PRS in general. It is, therefore, essential to include participants representing diverse populations in genomic medicine studies to ensure equitable benefit from scientific discoveries and to prevent further increase in health disparities.

To circumvent this, we perform combined analysis with additional cases and controls from the MVP to increase the gain in statistical power for novel locus discoveries in different ancestral groups. Although the most straightforward solution to increase PRS performance across ancestral groups is to build well-powered GWAS resources for diverse populations, we will entertain an alternative approach by taking advantage of the observation that genetic data from different ancestral groups still share substantial information, although with differences in allele frequency, linkage disequilibrium structure, and genetic architecture.

Recently, models have been developed to borrow information from European ancestral groups to help boost the power of predictions in non-European ancestral groups. We plan to conduct a comprehensive simulation and real data analyses in the MVP to evaluate these newly developed cross-population prediction models, including XPASS^19^ and PRScsx^20^ (that have been published), MePred and SDPRX (that are being developed by MVP researchers),^21^ and other methods. We believe this innovative comprehensive evaluation will offer both insights and guidance to improve risk stratifications for both European and non-European populations, expand our knowledge of disease mechanisms, and benefit disease screening and early prevention strategies.

## Authors Contribution

All authors have contributed to the writing, editing, and approval of this publication.

## Abbreviations Used

AFR: African
AUC: area under the receiver operating characteristic curve
GWAS: genome-wide association studies
MVP: Million Veteran Program
PRS: polygenic risk score
PRS313: 313-variant PRS

## References

[1] Harris R, Yeatts J, Kinsinger L. Breast cancer screening for women ages 50 to 69 years a systematic review of observational evidence. Prev Med 2011;53(3):108–114; doi: 10.1016/j.ypmed.2011.07.004 21820465

[2] Fisher B, Costantino JP, Wickerham DL, et al. Tamoxifen for the prevention of breast cancer: Current status of the National Surgical Adjuvant Breast and Bowel Project P-1 study. J Natl Cancer Inst 2005;97(22):1652–1662. 16288118 10.1093/jnci/dji372

[3] Cuzick J, Forbes J, Edwards R, et al. First results from the International Breast Cancer Intervention Study (IBIS-I): A randomised prevention trial. Lancet 2002;360(9336):817–824. 12243915 10.1016/s0140-6736(02)09962-2

[4] Kuhl CK. Predict, then act: Moving toward tailored prevention. J Clin Oncol 2019;37(12):943–945. 30844319 10.1200/JCO.19.00068

[5] Zhang H, Ahearn TU, Lecarpentier J, et al. Genome-wide association study identifies 32 novel breast cancer susceptibility loci from overall and subtype-specific analyses. Nat Genet 2020;52(6):572–581. 32424353 10.1038/s41588-020-0609-2 PMC7808397

[6] Jia G, Lu Y, Wen W, et al. Evaluating the utility of polygenic risk scores in identifying high-risk individuals for eight common cancers. JNCI Cancer Spectr 2020;4(3); doi: 10.1093/jncics/pkaa021 PMC7306192 32596635

[7] Yanes T, Young M-A, Meiser B, et al. Clinical applications of polygenic breast cancer risk: A critical review and perspectives of an emerging field. Breast Cancer Res 2020;22(1); doi: 10.1186/s13058-020-01260-3 PMC7026946 32066492

[8] Houlahan KE. Do breast cancer risk scores work for you? J Natl Cancer Inst 2021;113(9):1118–1119. 33769538 10.1093/jnci/djab052

[9] Minnier J, Rajeevan N, Gao L, et al. Polygenic breast cancer risk for women veterans in the million veteran program. JCO Prec Oncol 2021;(5):1178–1191; doi: 10.1200/po.20.00541 PMC8345920 34381935

[10] Mavaddat N, Michailidou K, Dennis J, et al. Polygenic risk scores for prediction of breast cancer and breast cancer subtypes. Am J Hum Genet 2019;104(1):21–34. 30554720 10.1016/j.ajhg.2018.11.002 PMC6323553

[11] Choudhury PP, Maas P, Wilcox A, et al. iCARE: An R package to build, validate and apply absolute risk models. PLoS One 2020;15(2):e0228198; doi: 10.1371/journal.pone.0228198 32023287 PMC7001949

[12] Choudhury PP, Wilcox AN, Brook MN, et al. Comparative validation of breast cancer risk prediction models and projections for future risk stratification. J Natl Cancer Inst 2020;112(3):278–285; doi: 10.1093/jnci/djz113 31165158 PMC7073933

[13] Maas P, Barrdahl M, Joshi AD, et al. Breast cancer risk from modifiable and nonmodifiable risk factors among white women in the United States. JAMA Oncol 2016;2(10):1295–1302. 27228256 10.1001/jamaoncol.2016.1025 PMC5719876

[14] Shieh Y, Fejerman L, Lott PC, et al. A polygenic risk score for breast cancer in US Latinas and Latin American women. J Natl Cancer Inst 2020;112(6):590–598; doi: 10.1093/jnci/djz174 31553449 PMC7301155

[15] Du Z, Gao G, Adedokun B, et al. Evaluating polygenic risk scores for breast cancer in women of African Ancestry. J Natl Cancer Inst 2021;113(9):1168–1176. 33769540 10.1093/jnci/djab050 PMC8418423

[16] Liu C, Zeinomar N, Chung WK, et al. Generalizability of polygenic risk scores for breast cancer among women with European, African, and Latinx Ancestry. JAMA Netw Open 2021;4(8):e2119084. 34347061 10.1001/jamanetworkopen.2021.19084 PMC8339934

[17] DeSantis CE, Ma J, Gaudet MM, et al. Breast cancer statistics, 2019. CA Cancer J Clin 2019;69(6):438–451. 31577379 10.3322/caac.21583

[18] Adedokun B, Du Z, Gao G, et al. Cross-ancestry GWAS meta-analysis identifies six breast cancer loci in African and European ancestry women. Nat Commun 2021;12(1):4198–4205. 34234117 10.1038/s41467-021-24327-x PMC8263739

[19] Cai M, Xiao J, Zhang S, et al. A unified framework for cross-population trait prediction by leveraging the genetic correlation of polygenic traits. Am J Hum Genet 2021;108(4):632–655. 33770506 10.1016/j.ajhg.2021.03.002 PMC8059341

[20] Ruan Y, Lin Y-F, Feng Y-CA, et al. Improving polygenic prediction in ancestrally diverse populations. Nat Genet 2022;54(5):573–580. 35513724 10.1038/s41588-022-01054-7 PMC9117455

[21] Zhou G, Chen T, Zhao H. SDPRX: A statistical method for cross-population prediction of complex traits. Am J Hum Genet 2023;110(1):13–22. 36460009 10.1016/j.ajhg.2022.11.007 PMC9892700

